# Genomic imbalances in precancerous tissues signal oral cancer risk

**DOI:** 10.1186/1476-4598-8-50

**Published:** 2009-07-23

**Authors:** Cathie Garnis, Raj Chari, Timon PH Buys, Lewei Zhang, Raymond T Ng, Miriam P Rosin, Wan L Lam

**Affiliations:** 1 Department of Cancer Genetics and Developmental Biology; British Columbia Cancer Research Centre, Vancouver, BC, V5Z 1L3, Canada; 2 Department of Pathology and Laboratory Medicine, University of British Columbia, Vancouver, BC, V6T 2B5, Canada; 3 Division of Oral Biological and Medical Sciences, Faculty of Dentistry, University of British Columbia, Vancouver, BC, V6T 1Z3, Canada; 4 British Columbia Oral Cancer Prevention Program, British Columbia Cancer Agency, Vancouver, BC V5Z 4E6, Canada; 5 Department of Computer Science, University of British Columbia, Vancouver, B.C., V6T 1Z4, Canada

## Abstract

Oral cancer develops through a series of histopathological stages: through mild (low grade), moderate, and severe (high grade) dysplasia to carcinoma *in situ *and then invasive disease. Early detection of those oral premalignant lesions (OPLs) that will develop into invasive tumors is necessary to improve the poor prognosis of oral cancer. Because no tools exist for delineating progression risk in low grade oral lesions, we cannot determine which of these cases require aggressive intervention. We undertook whole genome analysis by tiling-path array comparative genomic hybridization for a rare panel of early and late stage OPLs (n = 62), all of which had extensive longitudinal follow up (>10 years). Genome profiles for oral squamous cell carcinomas (n = 24) were generated for comparison. Parallel analysis of genome alterations and clinical parameters was performed to identify features associated with disease progression. Genome alterations in low grade dysplasias progressing to invasive disease more closely resembled those observed for later stage disease than they did those observed for non-progressing low grade dysplasias. This was despite the histopathological similarity between progressing and non-progressing cases. Strikingly, unbiased computational analysis of genomic alteration data correctly classified nearly all progressing low grade dysplasia cases. Our data demonstrate that high resolution genomic analysis can be used to evaluate progression risk in low grade OPLs, a marked improvement over present histopathological approaches which cannot delineate progression risk. Taken together, our data suggest that whole genome technologies could be used in management strategies for patients presenting with precancerous oral lesions.

## Background

At present, risk of progression in oral premalignant lesions (OPLs) is typically determined based on histopathological evaluation of biopsied material. High grade dysplasia (HGD) and carcinoma *in situ *(CIS) are considered high risk for progression to invasive disease. In contrast, only a small proportion of low grade dysplasias (LGDs) – which represent the majority of diagnosed OPLs – progress to invasive disease [1,2]. Histological features cannot currently be used to delineate "progressing" and "non-progressing" LGDs [3]. Consequently, LGDs that are prime candidates for early intervention are not easily identified. Novel approaches for defining progression likelihood for histopathologically similar LGDs are required.

Chromosome instability, particularly loss of chromosome arms 3p and 9p, has previously been associated with an increased probability of progression in oral cancer, demonstrating the potential utility of molecular markers in predicting progression risk [4-7]. Additionally, p53 status has been used to predict progression in Barretts esophagus and other groups have reported genomic instability in tumor-associated dysplastic oral tissue [8-11]. To date, efforts to undertake whole genome analysis of premalignant lesions have been precluded by 1) the rarity of LGD specimens with longitudinal follow-up and clinical outcome details and 2) the lack of robust high resolution genome profiling methodologies that can utilize the limited DNA yield from microdissected formalin-fixed paraffin-embedded lesions. In this study, we compared the genomes of precancerous oral tissues from different disease stages to identify stage-specific DNA alterations. Analysis of this rare sample set not only revealed qualitative and quantitative differences in DNA alterations depending on histopathological stage, but also showed that these features are associated with known clinical outcomes.

## Results and discussion

**Figure 1:**
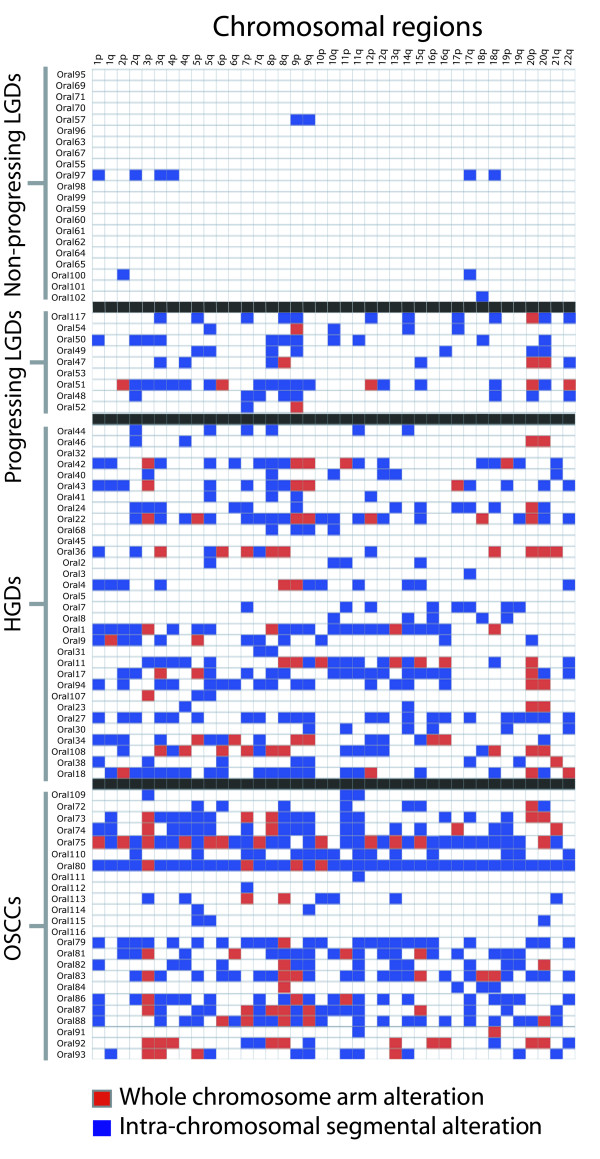
**Summary of chromosomal alterations for all 86 cases**. Samples are grouped into non-progressing low grade dysplasias, progressing low grade dysplasias, high grade lesions (severe dysplasia and CIS lesions), and oral squamous cell carcinomas. A blue box indicates the presence of at least one segmental change on the chromosome arm and a red box represents a whole arm alteration. Copy number changes due to polymorphic regions were not included in the analysis. Case numbers are listed to the left, while chromosome arms are listed at the top.

**Figure 2:**
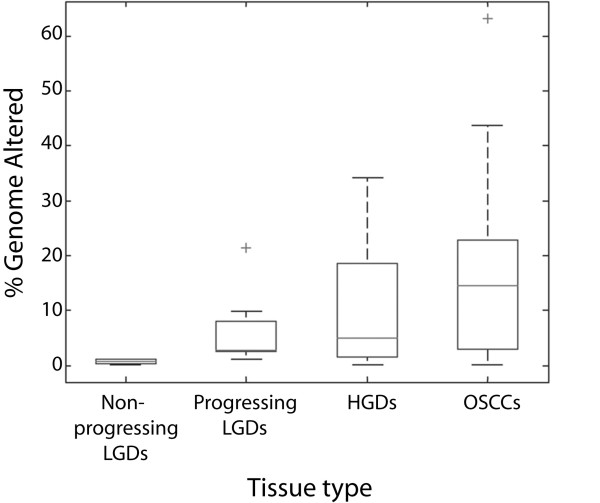
**Box plot showing percentage of genome altered**. As in Figure 1, samples are grouped into non-progressing LGDs, progressing LGDs, HGD/CIS lesions, and OSCCs. Genome altered was calculated by dividing the number of clones deemed changed (gain or loss), by *aCGH-Smooth*, by the total number of clones assayed for each sample. Chromosomes X and Y were excluded from this analysis.

Genome profiles were generated by tiling-path array CGH for a panel of 86 oral lesions with longitudinal follow-up that included 24 invasive oral squamous cell carcinomas (OSCCs) and 62 OPLs. This sample panel was comprised of 32 HGD and CIS lesions, 21 non-progressing LGDs, and nine progressing LGDs where the average time to progression to a higher grade was 27.2 months. (Demographic patient information are supplemental – see Additional File 1: Table S1.) Two classes of segmental changes were defined: whole chromosome arm changes and segmental DNA copy number changes. Segmental genomic gains and losses were defined using the *aCGH-Smooth *algorithm (Figure 1) [12]. Similar to earlier findings using locus specific probes, both Figure 1 and Figure 2 show how increases in lesion severity paralleled increases in the degree of genomic instability (i.e. the number of genomic alterations) [13,14].

HGDs, despite being classified as pre-invasive, showed a degree of genomic instability comparable to what was observed in invasive tumors; they had an average of 16.6 segmental DNA changes and 2.4 whole chromosome arm changes per sample, while tumors averaged 23.2 segmental DNA changes and 3.5 whole arm changes. Interestingly, the most frequently observed regions of genomic alteration were different for HGD/CIS lesions as compared to invasive tumors. In terms of whole chromosome arm changes, the most commonly observed alteration in HGD/CIS lesions was gain of chromosome 20p (10/32 cases). On the other hand, deletion of chromosome 3p (11/27) and gain of chromosome 8q (9/27) were the most commonly observed whole arm alterations seen in invasive tumors. With respect to intra-chromosomal segmental DNA copy number changes, the most commonly altered regions in HGD/CIS lesions occurred within chromosome arms 1p, 2q, 3q, 5q, 7q, and 8p, while the most common changes seen for invasive tumors occurred within chromosome arms 5p, 9q, 11q, and 19p (Figure 1). This may indicate that the genetic alterations that drive disease initiation and progression are different from those that drive tumor invasion, the earlier alterations masked by subsequent genomic instability.

**Figure 3:**
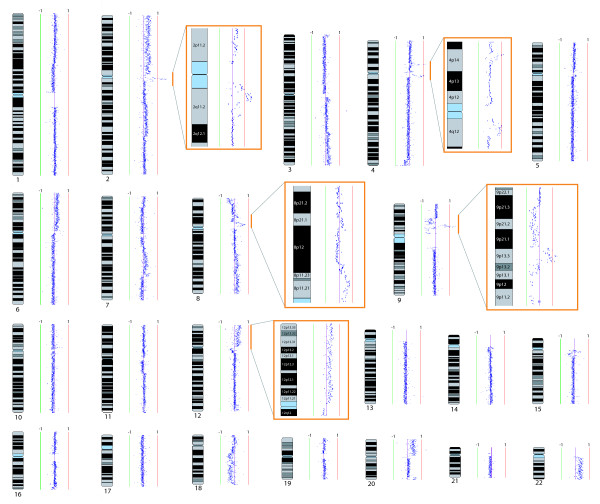
**Whole genome tiling path array CGH karyogram of an oral low grade dysplasia which subsequently progressed to cancer**. Whole genome tiling path array CGH karyogram of an oral low grade dysplasia which subsequently progressed to cancer (Oral51). Each dark blue dot on the karyogram represents the average signal ratio for an individual BAC clone from the array. Clones were plotted vertically against known chromosomal position. Log2 signal intensity ratios for each clone were plotted horizontally, with colored vertical lines denoting log2 signal ratios from -1 to 1. Where the signal intensity ratio equals zero (purple line), equivalent DNA copy number between the sample and the reference DNA was inferred. Alternatively, DNA copy number increases were inferred where log2 > 0 (red line) and losses were inferred where log2 < 0 (green line). Numerous whole chromosome, whole arm, and segmental changes are apparent. Examples of these alterations were magnified and are represented in orange boxes. High level segmental amplicons for chromosome 2 and 4 are depicted in this manner. Lower copy number segmental (chromosome 8) and whole arm (chromosome 12) gains are similarly shown. The magnified image for Chromosome 9 shows a complex genomic rearrangement that includes multiple segmental losses and a high level segmental amplification event.

Amongst low grade dysplasias, only some cases appeared to harbor genome alterations. Review of the clinical data revealed a striking association between the presence of genomic imbalances and subsequent progression to invasive disease. Figure 3 shows a typical karyogram of a progressing LGD, where multiple alterations are apparent. Across the progressing LGD cases, an average of 9.2 genomic changes was observed (either intra-chromosomal or whole arm alterations). Interestingly, no alterations were shared by all progressing LGD cases. Chromosome arm 9p was the most frequently altered chromosome (altered in 78% of cases), followed by 8q, 20p, and 20q (each observed in 56% of cases). Of the 21 non-progressing LGDs, alterations were only detected in four cases – and in these instances, only one or two changes were typically observed.

The total genomic alterations for progressing LGDs more closely resembled the HGD cases, which are known to have a higher likelihood of progression (Figure 2) [15,16]. These data suggest that the degree of genomic instability in a given OPL may have utility for predicting progression likelihood. To investigate this further, we used patterns of genomic alteration (Figure 1) to classify individual lesions relative to the rest of the sample panel. We first evaluated whether DNA alteration features could be used to differentiate lesions with a high risk of progression and tumors (HGD, CIS lesions, OSCCs) from lesions that did not progress (non-progressing LGD lesions). Specifically, we used a k-nearest neighbor statistical analysis (where k = 3; see Methods) to test if these very different groups could be distinguished based on the number of DNA alterations they harbored (with whole chromosome arm and segmental changes weighted equally) [17]. Each sample was called as either "HGD/CIS/OSCC" or "non-progressing LGD" based on a consensus comparison against the three closest samples, as defined by a Euclidean distance calculated using the number of DNA alterations (the histological subclass was known for all samples except the given test case). If multiple samples were of equal distance from the unclassified sample, then all of the samples at that distance were used. For example, if five samples shared the third closest distance, a total of seven samples would be used in deriving the consensus classification (the first two nearest neighbors, plus the five neighbors with equal distance). This blind approach, based solely on analysis of genome alterations, correctly classified most of the samples we analyzed, including 81% of the LGDs and 87.5% of the HGD/CIS/OSCC lesions. Moreover, the difference in the number of segmental alterations between non-progressing LGDs and HGD/CIS/OSCC was statistically significant (Mann Whitney U test, *p *< 10^-12^).

Given our earlier observation that the genome features of progressing LGDs more closely resembled higher grade lesions than non-progressing LGDs, we next checked to see if patterns of DNA alteration could be used to distinguish between progressing and non-progressing LGDs. To do this, we applied the same k-nearest neighbor approach described above. Briefly, we analyzed each progressing LGD genome profile against all of the genome profiles generated for the non-progressing LGDs and the HGD/CIS/OSCC lesions. (DNA alterations were defined and weighted as above.) If the progressing lesion more closely resembled the high grade lesions and tumors, it was classified as a progressing LGD. If it more closely resembled the non-progressing LGDs, it was grouped with those cases. This approach correctly classified 88.9% of the progressing LGD lesions and the difference in the number of segmental alterations between non-progressing LGDs and progressing LGDs was also statistically significant (*p *< 10^-4^). This rate of successful classification stands as a marked improvement over current histopathological approaches, which are not at all able to predict progression likelihood for LGDs.

## Conclusion

This study provides the first detailed analysis of the genomes of oral premalignant lesions and supports the use of genome profiling as a clinical tool for predicting progression risk. The recent association of chromosomal instability with post-resection oral tumor recurrence also supports application of genomic tools for guiding management strategies [18]. Comparative analysis of the genome profiles of these early stage specimens revealed a conspicuous difference in the abundance of genetic alterations between low grade dysplasias that progressed to invasive disease and those that did not. The genome profiles of low grade OPLs known to progress to cancer more closely resembled profiles of high grade OPLs than they did those of non-progressing low grade OPLs. This was despite the histopathological similarity between progressing and non-progressing cases. As with the higher grade lesions, progressing low grade OPLs showed complex patterns of genome alteration. These alterations included both gross chromosomal aberrations, which include whole arm and whole chromosome amplifications and deletions, as well as localized intra-chromosomal segmental gains and losses (alterations in some instances that would have been too small to detect by conventional molecular cytogenetic techniques). These findings, combined with previous reports linking loss of heterozygosity status to disease progression, demonstrate that there are multiple genetic mechanisms involved in progression to invasive oral cancer. More importantly, they show that characterization of genomic alterations in low grade dysplasias can be used as an effective predictor for disease progression likelihood.

## Methods

### Tissue samples

Formalin-fixed paraffin-embedded tissue blocks were obtained from the British Columbia Oral Biopsy Service and diagnoses were confirmed by an oral pathologist. Cells of the OPLs and tumor were microdissected from H&E-stained sections. Only samples with greater than 80% tumor cell content were used in this study. Microdissected tissue was placed in a sodium dodecyl sulfate solution with proteinase K at 48°C and spiked with additional enzyme twice a day for 72 hours. Genomic DNA was extracted by a standard phenol:chloroform/ethanol precipitation protocol. Samples were selected for further study based on the quantity and quality of DNA. Demographic information for these samples can be viewed in Additional File 1: Table S1. All lesions were obtained from different patients and were taken before treatment was given.

### Whole genome tiling-path array comparative genomic hybridization (CGH)

The array platform, comprised of 26,363 overlapping elements, was manufactured on site, as previously described [19,20]. Briefly, 200 ng of test and reference DNA were separately labeled with Cyanine-3 and Cyanine-5 dCTPs Using the BioPrime DNA labeling system (Invitrogen, Burlington, Ontario, Canada). DNA probes were then pooled and unincorporated nucleotides were removed with a YM-30 Microcon centrifugation tube (Millipore). Next, 100 μg of Cot-1 DNA (Invitrogen) was added and the entire mixture was precipitated. This material was then re-suspended in a 45 μl cocktail consisting of DIG Easy hybridization solution (Roche), sheared herring sperm DNA (Sigma-Aldrich), and yeast tRNA (Calbiochem). Probe denaturing and blocking steps followed at 85°C and 45°C for 10 minutes and for one hour respectively. Subsequently, the probe mixture was applied to the surface of the array, coverslips were applied, and arrays were incubated at 45°C for 36 hours. Slides next underwent five agitating washes in 0.1× saline sodium citrate, 0.1% SDS at 45°C (each wash ~5 min). Rinses with 0.1× SSC followed, then drying by centrifugation. Genome profiles are available online through the NCBI Gene Expression Omnibus (, GSE11275).

### Imaging and analysis

A CCD-based imaging system (Arrayworx eAuto, API, Issaquah, WA) was used to determine signal intensities in each dye channel. Images were analyzed with Softworx array analysis software. Experimental bias due to the array platform was removed using a stepwise normalization framework [21]. All spots with standard deviations > 0.1 were excluded from the analysis. Custom viewing software, *SeeGH*, was used to visualize all data as log_2 _ratio plots [22]. Array probes were aligned based on the May 2004 mapping of the human genome. To delineate regions of copy number gain and loss, segmentation analysis was performed on array data using the *aCGH-Smooth *algorithm (default parameters were used on all settings except for the following changes: lambda value = 6.75, maximum number of breakpoints in initial pool = 100, minimum difference between levels = 0.2) [12]. The copy number status for clones filtered by the above criteria was inferred by the status of neighboring clones. A chromosome arm was considered changed if ≥ 90% of clones spanning the arm exhibited the same alteration status. A segmental change was defined as being a continuous alteration between 10 clones and half a chromosome arm in size. Segmental alterations reported to be polymorphisms were excluded from analysis [23]. Tumor genome profile data are available online through the NCBI Gene Expression Omnibus (, accession number GSE11275).

### Statistical analysis

K-nearest neighbor analysis was used to classify the samples based on the number of segmental and whole chromosome arm changes that were present. Since an even number of groups existed, we used the nearest three neighbors. A standard Euclidean distance metric was used to determine the distance to each neighbor, with equal weighting given to both segmental and whole arm changes. If more than three neighbors were at equal distance, all neighbors at that distance were used.

## Abbreviations

OPL: oral premalignant lesion; HGD: high grade dysplasia; LGD: low grade dysplasia; CIS: carcinoma *in situ*; OSCC: oral squamous cell carcinoma; CGH: comparative genomic hybridization.

## Competing interests

The authors declare that they have no competing interests.

## Authors' contributions

CG, RC, TPHB, and RTN undertook experiments and data analysis. LZ provided expert pathological review. MPR and WLL were principal investigators on this project. All authors read, provided critical feedback, and approved the final manuscript.

## Supplementary Material

Additional file 1 **Supplemental table**. Demographic patient information. Click here for file
